# Inference of Genetic Networks from Pseudo Time Series of Single-cell Gene Expression Data using Modified Random Forests

**DOI:** 10.1007/s11538-026-01612-8

**Published:** 2026-02-21

**Authors:** Shuhei Kimura, Ryosuke Misaki, Masato Tokuhisa, Keita Iida, Mariko Okada

**Affiliations:** 1 Faculty of Engineering, Tottori University, 4-101, Koyama-minami, Tottori, 680-8552 Japan; 2 Avian Infectious Disease and Global Health Research Center, Tottori University, 4-101, Koyama-minami, Tottori, 680-8553 Japan; 3 Graduate School of Sustainability Sciences, Tottori University, 4-101, Koyama-minami, Tottori, 680-8552 Japan; 4 Institute for Protein Research, Osaka University, 3-2, Yamadaoka, Suita, 565-0871 Japan

**Keywords:** scRNA-seq, Genetic network, GENIE3, Pseudo time-series data

## Abstract

This study proposes a novel method for inferring genetic networks using both steady-state and pseudo time-series data of single-cell gene expressions. While several methods for inferring genetic networks from time series of bulk-cell gene expression data have been proposed, many of these approaches use time derivatives of gene expression levels. However, since pseudo time-series data lack precise temporal information about when measurements were taken, time derivatives cannot be calculated from this data. Therefore, existing methods are ineffective for analyzing pseudo time-series data. To address this limitation, our proposed method does not use time derivatives of gene expression levels but uses their signs. We theorize that, even when no precise temporal information is available, the signs of time derivatives, which indicate whether the gene expression levels are increasing or decreasing, can be estimated from pseudo time-series data. Our approach was designed on the basis of GENIE3 and its extensions, which, although essentially intended to infer genetic networks from bulk-cell gene expression data, have reportedly performed well in this respect. Validation through numerical experiments with both artificial and real gene expression data confirms the effectiveness of our proposed method.

**Supplementary Information:**

The online version contains supplementary material available at 10.1007/s11538-026-01612-8.

## Introduction

High-throughput technologies such as RNA-seq using next-generation sequencers make it possible to measure the expression levels of tens of thousands of genes. To extract useful information from these data, researchers have increasingly focused on the use of inferred genetic networks that represent abstract regulatory relationships among genes. Inferred models of genetic networks can help biologists generate hypotheses and facilitate the design of experiments. A number of genetic network inference methods have been proposed (Chou and Voit 2009; Nguyen et al. 2020; Saint-Antoine and Singh 2020).

While most inference methods were originally designed to analyze bulk-cell gene expression data, single-cell resolution data have recently become available. Methods that analyze bulk-cell steady-state data are also capable of analyzing single-cell steady-state data. However, due to high noise levels in single-cell measurements, methods designed for bulk-cell time-series data often fail to reliably infer genetic networks from single-cell time-series data. Single-cell time-series datasets measured in different series of experiments often exhibit qualitatively different trends.

In single-cell data analysis, pseudo time-series of gene expression levels can serve as an alternative to time-series data. We obtain these pseudo time-series data through pseudo-temporal ordering analysis (Trapnell et al. 2014), a method of arranging individual cell measurements based on their similarities. While pseudo time-series data resemble conventional time-series data, they lack precise temporal information about when the measurements were taken. Since inference methods designed for bulk-cell time-series data typically require information on measurement timing, they are ineffective in analyzing pseudo time-series data. One existing inference method (Matsumoto et al. 2017) attempts to analyze pseudo time-series data by implicitly assuming that pseudotime defined by the pseudo-temporal ordering analysis correlates linearly with actual time. However, since this assumption may not always hold, we cannot rely on networks inferred through this approach.

This study proposes a novel method capable of inferring genetic networks from both pseudo time-series and steady-state data of single-cell gene expressions (Kimura et al. 2023). While many existing methods capable of analyzing bulk-cell time-series data rely on time derivatives of gene expression levels to infer genetic networks, our proposed method uses their signs. As noted above, the pseudo time-series data consist of individual cell measurements ordered along the progression of a cellular process but lack precise information about measurement times. Consequently, we cannot estimate time derivatives of gene expression levels from such data. However, we theorize that the signs of the time derivatives of the gene expression levels can be estimated, even when no precise temporal information is available. Our proposed method was designed based on GENIE3 (Huynh-Thu et al. 2010) and one of its extensions (Kimura et al. 2019), algorithms originally intended to infer genetic networks from bulk-cell gene expression data (Huynh-Thu et al. 2010; Huynh-Thu and Geurts 2018; Kimura et al. 2019; Maduranga et al. 2013; Petralia et al. 2015) but known for performing well. We evaluate the performance of our proposed inference method through numerical experiments with artificial and real genetic network inference problems.

## Random-Forest-Based Inference Method

The proposed method is modeled on the random-forest-based inference method (Kimura et al. 2019), which is an extension of GENIE3 (Huynh-Thu et al. 2010). The method proposed in this study repeatedly runs GENIE3. Therefore, this section will first describe the random-forest-based inference method (Kimura et al. 2019) and then explain the relationship between the random-forest-based inference method and GENIE3.

### Model for Describing Genetic Networks

The random-forest-based inference method (Kimura et al. 2019) assumes that a genetic network can be represented by the following set of ordinary differential equations.

1 $$\begin{aligned} \frac{dX_n}{dt} = F_n\left( {\textbf {X}}_{-n}\right) - \beta _n X_n, \hspace{5mm}(n = 1, 2, \cdots , N), \end{aligned}$$

where $${\textbf {X}}_{-n} = (X_1, \cdots , X_{n-1}, X_{n+1}, \cdots , X_N)$$, $$X_m$$ is the expression level of the *m*-th gene, *N* is the number of genes contained in the target network, $$\beta _n$$ ($$ > 0$$) is a constant parameter, and $$F_n$$ is a function of arbitrary form.

In the method (Kimura et al. 2019), an inference problem for a genetic network consisting of *N* genes is decomposed into *N* subproblems, each corresponding to a single gene. By solving the *n*-th subproblem, the method obtains a reasonable approximation of the function $F_n$ and an appropriate value for the parameter $\beta _n$. Using this approximation, the method then computes confidence values for regulatory interactions affecting the *n*-th gene from the other genes. In the next section, we describe ways for solving the *n*-th subproblem and computing confidence values.

### Solving the *n*-th Subproblem

To obtain an approximation of $F_n$ and a value for $\beta _n$, the inference method (Kimura et al. 2019) employs a weighted least-squares method. Thus, the *n*-th subproblem is defined as an optimization problem for the following one-dimensional function.

2 $$\begin{aligned} S_n(\beta _n) = \sum _{k=1}^{K_T} \frac{w_k^T}{\beta _n} \left[ \left. \frac{dX_n}{dt} \right| _{t_k} - \hat{F}_n \left( \left. {\textbf {X}}_{-n} \right| _{t_k}; \beta _n \right) + \beta _n \left. X_n \right| _{t_k} \right] ^2 \nonumber \\ + \sum _{k=1}^{K_S} \frac{w_k^S}{\beta _n} \left[ \left. \frac{dX_n}{dt} \right| _{s_k} - \hat{F}_n \left( \left. {\textbf {X}}_{-n} \right| _{s_k}; \beta _n \right) + \beta _n \left. X_n \right| _{s_k} \right] ^2, \end{aligned}$$

where $\left. {\textbf {X}}_{-n}\right| _{t_k} = (\left. X_1\right| _{t_k}, \cdots , \left. X_{n-1}\right| _{t_k}, \left. X_{n+1}\right| _{t_k}, \cdots , \left. X_N\right| _{t_k})$, $\left. {\textbf {X}}_{-n}\right| _{s_k} = (\left. X_1\right| _{s_k},$ $\cdots , \left. X_{n-1}\right| _{s_k}, \left. X_{n+1}\right| _{s_k}, \cdots , \left. X_N\right| _{s_k})$, and $\left. X_m \right| _{t_k}$ and $\left. X_m \right| _{s_k}$ are the expression levels of the *m*-th gene at the *k*-th measurements of time-series and steady-state experiments, respectively. The terms $\left. \frac{dX_n}{dt} \right| _{t_k}$ and $\left. \frac{dX_n}{dt} \right| _{s_k}$ represent the time derivatives of the expression levels of the *n*-th gene at the *k*-th measurements of the time-series and steady-state experiments, respectively. We estimate $\left. \frac{dX_n}{dt} \right| _{t_k}$ values directly from the measured time-series of the gene expression levels using a smoothing technique. In contrast, all $\left. \frac{dX_n}{dt} \right| _{s_k}$ values are set to zero because the data were measured under steady-state conditions. $K_T$ ($\ge 2$) and $K_S$ ($\ge 0$) represent the numbers of measurements performed in the time-series and steady-state experiments, respectively. $w_k^T$ and $w_k^S$ are weight parameters for the *k*-th measurements in the time-series and steady-state experiments, respectively. Previous studies have shown that the performance of the random-forest-based inference method improves when appropriate values are assigned to the parameters $w_k^T$ and $w_k^S$ (Kimura et al. 2019). A technique for determining these values using measurement data has been proposed (Kimura et al. 2022a).

$\hat{F}_n \left( \hspace{1mm}\cdot \hspace{1mm}; \beta _n \right)$ is an approximation of function $F_n$ trained under a given $\beta _n$. To compute a value for the objective function (2), we must obtain an approximation of function $F_n$, i.e., $\hat{F}_n$. As the approximation of function $F_n$, the inference method (Kimura et al. 2019) uses a random forest (Breiman 2001) trained on training data consisting of the following input-output pairs,

$$\begin{aligned} \left\{ \left. \left( \left. {\textbf {X}}_{-n} \right| _{t_k}, \left. \frac{dX_n}{dt} \right| _{t_k} + \beta _n \left. X_n \right| _{t_k} \right) \right| k = 1, 2, \cdots , K_T \right\} \\ \cup \left\{ \left. \left( \left. {\textbf {X}}_{-n} \right| _{s_k}, \left. \frac{dX_n}{dt} \right| _{s_k} + \beta _n \left. X_n \right| _{s_k} \right) \right| k = 1, 2, \cdots , K_S \right\} . \end{aligned}$$

We should note here that, when training the random forest, the method (Kimura et al. 2019) incorporates the weight parameters $w_k^T$ and $w_k^S$ to maintain consistency with the objective function (2). The training data contain the parameter $\beta _n$, whose value must be determined. However, when computing a value for the objective function (2), a value for parameter $\beta _n$ is always provided. The random-forest-based inference method (Kimura et al. 2019) uses the golden section search method (Press et al. 1995) to minimize the function (2). dynGENIE3 (Huynh-Thu and Geurts 2018), another extension of GENIE3, was designed based on the idea similar to that of this method. While the random-forest-based inference method estimates a value for $\beta _n$ through the optimization of the function (2), however, dynGENIE3 requires its value in advance.

By analyzing the random forest obtained through the optimization of the function (2), the inference method (Kimura et al. 2019) computes confidence values for regulations of the *n*-th gene from the other genes. For this purpose, the method uses the standard variable importance measure, which computes importance scores based on the total reduction in output value variance due to the split (Breiman 2001).

### Relation to GENIE3

GENIE3, proposed by Huynh-Thu and colleagues, was the first method to use random forests for genetic network inference (Huynh-Thu et al. 2010). GENIE3 uses steady-state gene expression data to infer genetic networks. The random-forest-based inference method (Kimura et al. 2019) described above was designed based on GENIE3.

When only steady-state data is available, the *n*-th differential equation from equations (1), which corresponds to the *n*-th gene, can be written as

3 $$\begin{aligned} \left( \frac{dX_n}{dt} = \right) 0 = F_n\left( {\textbf {X}}_{-n}\right) - \beta _n X_n. \end{aligned}$$

By dividing both sides of equation (3) by $\beta _n$ and replacing $F_n\left( {\textbf {X}}_{-n}\right) /\beta _n$ with $G_n\left( {\textbf {X}}_{-n}\right)$, we obtain

4 $$\begin{aligned} 0 = G_n\left( {\textbf {X}}_{-n}\right) - X_n. \end{aligned}$$

As mentioned in the previous section, the inference method (Kimura et al. 2019) computes confidence values for regulations of the *n*-th gene from other genes by analyzing the approximation of function $F_n$. Since function $F_n$ multiplied by a constant yields function $G_n$, we can also obtain confidence values by analyzing a good approximation of function $G_n$. GENIE3 obtains the approximation of function $G_n$ by training a random forest based on the following input-output pairs

$$ \left\{ \left. \left( \left. {\textbf {X}}_{-n} \right| _{s_k}, \left. X_n \right| _{s_k} \right) \right| k= 1,2, \cdots , K_S \right\} . $$

As described above, GENIE3 uses no time-series data when inferring genetic networks. Therefore, GENIE3 can analyze single-cell gene expression data that have been measured under steady-state conditions.

## Inference Method using Pseudo Time-Series Data

In this study, we propose a novel method for inferring genetic networks from pseudo time-series and steady-state single-cell gene expression data (Kimura et al. 2023). Similar to the random-forest-based inference method (Kimura et al. 2019) described in the previous section, our proposed method decomposes the inference problem of a genetic network consisting of *N* genes into *N* subproblems, each associated with a specific gene. The remainder of this section focuses on the *n*-th subproblem corresponding to the *n*-th gene.

As described previously, the proposed method uses the signs of the time derivatives of gene expression levels to infer genetic networks. The *n*-th subproblem thus contains the following labeled data,

$$ \left\{ \left. \left( \left. {\textbf {X}} \right| _{pt_k}, \left. Y_n \right| _{pt_k} \right) \right| k = 1, 2, \cdots , K_{PT} \right\} \cup \left\{ \left. \left( \left. {\textbf {X}} \right| _{s_k}, \left. Y_n \right| _{s_k} \right) \right| k = 1, 2, \cdots , K_S \right\} , $$

where $K_{PT}$ and $K_S$ represent the numbers of measurements (cells) in the pseudo time-series and steady-state data, respectively. $\left. {\textbf {X}} \right| _{pt_k} = (\left. X_1\right| _{pt_k}, \left. X_2\right| _{pt_k}, \cdots , \left. X_N\right| _{pt_k})$ and $\left. {\textbf {X}}\right| _{s_k} = (\left. X_1\right| _{s_k},$ $\left. X_2\right| _{s_k},$ $\cdots , \left. X_N\right| _{s_k})$ represent the gene expression profiles for the *k*-th measurement in the pseudo time-series and steady-state datasets, respectively, where $\left. X_m \right| _{pt_k}$ and $\left. X_m \right| _{s_k}$ denote the expression levels of the *m*-th gene at the *k*-th measurement. $\left. Y_n \right| _{pt_k}$ and $\left. Y_n \right| _{s_k}$ represent class labels indicating the signs of the time derivatives of the *n*-th gene’s expression level at the *k*-th measurement in the pseudo time-series and steady-state data, respectively. Since $\left. {\textbf {X}} \right| _{s_k}$ values are measured under steady-state conditions in this study, all $\left. Y_n \right| _{s_k}$ values are set to ‘0’. In contrast, $\left. Y_n \right| _{pt_k}$ is assigned a label of ‘+’, ‘−’ or ‘0’ based on the sign of the time derivative of the *n*-th gene’s expression level at the *k*-th measurement in the pseudo time-series data. In addition, we do not use several measurements for inferring a genetic network. We assign a label of ‘X’ to each of these measurements. The procedure for assigning class labels to pseudo time-series data is detailed in Sect. 4.

For clarity of explanation, we divide the dataset for the *n*-th subproblem described above into four subsets: $D_n^+$, $D_n^-$, $D_n^0$ and $D_n^X$, which consist only of measurements labeled ‘+’, ‘−’, ‘0’ and ‘X’, respectively. Note that the proposed method does not use the measurements in $D_n^X$ for solving the *n*-th subproblem.

### Concept

The proposed method assumes that genetic networks can be described by a set of differential equations (1). Following the approach in Sect. 2.3, we focus on the *n*-th equation, which corresponds to the *n*-th gene in this model. By dividing both sides of this equation by $\beta _n$ and replacing $F_n\left( {\textbf {X}}_{-n}\right) /\beta _n$ with $G_n\left( {\textbf {X}}_{-n}\right)$, we obtain

5 $$\begin{aligned} \frac{dX_n}{dt}\Bigg /\beta _n = G_n\left( {\textbf {X}}_{-n}\right) - X_n. \end{aligned}$$

Since $\beta _n$ has a positive value, equation (5) suggests that, if we know the function $G_n$, we can infer the sign of the time derivative of the *n*-th gene’s expression level from the expression levels of all genes. Specifically, when the value of $G_n\left( {\textbf {X}}_{-n}\right) - X_n$ is positive, negative, or zero, we can conclude that the sign of the time derivative of the *n*-th gene is positive, negative, or zero, respectively. As noted in Sect. 2.3, GENIE3 (Huynh-Thu et al. 2010) can be used to approximate the function $G_n$ from steady-state gene expression data. This means we can estimate the signs of gene expression time derivatives using GENIE3. If the estimated signs are consistent with many training examples in datasets $D_n^+$ and $D_n^-$, therefore, the approximation of the function $G_n$ would be reasonable. In other words, we can use the training examples in datasets $D_n^+$ and $D_n^-$ to quantify the goodness of the approximation of the function $G_n$.

Our proposed method leverages this idea to infer genetic networks from steady-state and pseudo time-series single-cell gene expression data. Specifically, using datasets $D_n^+$ and $D_n^-$, our method tries to construct a more reasonable approximation of the function $G_n$ than that directly obtained by GENIE3.

### Algorithm

In this study, we propose a new inference method based on the concept introduced in Sect. 3.1. For the *n*-th subproblem, the proposed method runs GENIE3 using the dataset $D_n^0$ to obtain a random forest approximating the function $G_n$. This random forest comprises multiple regression trees, each of which also approximates function $G_n$. From this initial random forest, the method selects the regression tree most consistent with the examples in datasets $D_n^+$ and $D_n^-$, and discards the others. By repeating the above procedure, the method constructs a tree ensemble consistent with datasets $D_n^+$, $D_n^-$ and $D_n^0$. Since the resulting tree ensemble has an identical structure to a random forest, we can compute confidence values for regulations of the *n*-th gene from the other genes by analyzing this ensemble.

The following procedure describes how the proposed method solves the *n*-th subproblem corresponding to the *n*-th gene and computes the confidence values for regulations of the *n*-th gene from the other genes.

#### Step 1: Initialization

Receive input data in the form of pairs of single-cell gene expression data and their class labels, i.e., $D_n^+ \cup D_n^- \cup D_n^0$. As mentioned in Sect. 2, an earlier study (Kimura et al. 2019) showed that assigning appropriate weight parameters can improve the performance of the existing random-forest-based inference method. Accordingly, the proposed inference method incorporates weight parameters and requires their values as input. In this study, $w_k^+$, $w_k^-$ and $w_k^0$ denote the weight parameters assigned to the *k*-th measurements in datasets $D_n^+$, $D_n^-$ and $D_n^0$, respectively. These values can be determined using the established technique (Kimura et al. 2022a). This weighting approach has been introduced in order to prevent the performance degradation caused by the measurements similar to each other. If the user does not wish to use weighting, each parameter can simply be set to 1.0.

#### Step 2: Sampling from $D_n^0$

Randomly sample $|D_n^0|$ examples with replacement from the dataset $D_n^0$. This step comes from a bagging, a technique used in a training algorithm for random forests. Note here that, as described in the previous step, the weight values are assigned to all of the examples. Along with the examples, thus, get their weight values in this step.

#### Step 3: Execution of GENIE3

Run GENIE3 (Huynh-Thu et al. 2010) using training examples labeled ‘0’. Unlike the original GENIE3 algorithm, which randomly selects examples with replacement for each regression tree from the provided training data, our method trains all trees using the same set of examples selected in Step 2. Additionally, while the original GENIE3 does not consider weight parameters, the implementation in this study incorporates the weight parameters $w_k^0$ obtained in Step 2 when analyzing the given data. We denote the number of trees in the GENIE3 random forest as $N_{subtree}$.

#### Step 4: Sampling from $D_n^+$ and $D_n^-$

Randomly sample $|D_n^+|$ and $|D_n^-|$ examples with replacement from datasets $D_n^+$ and $D_n^-$, respectively. Similar to Step 2, get the weight values along with the examples. This study denotes the datasets constructed here from $D_n^+$ and $D_n^-$ as $D_n^{+*} = \left\{ \left. \left( \left. {\textbf {X}} \right| _k^{+*}, \left. Y_n \right| _k^{+*} \right) \right| k = 1, 2, \cdots , |D_n^+| \right\}$ and $D_n^{-*} =$ $\big \{ \left. \left( \left. {\textbf {X}} \right| _k^{-*}, \left. Y_n \right| _k^{-*} \right) \right| k = 1, 2, \cdots , |D_n^-| \big \}$, respectively, where $\left. {\textbf {X}} \right| _k^{+*} = ( \left. X_1\right| _k^{+*}, \left. X_2\right| _k^{+*},$ $\cdots , \left. X_N\right| _k^{+*})$ and $\left. {\textbf {X}} \right| _k^{-*} = ( \left. X_1\right| _k^{-*}, \left. X_2\right| _k^{-*}, \cdots , \left. X_N\right| _k^{-*})$. Further, $\left. X_m\right| _k^{+*}$ and $\left. X_m\right| _k^{-*}$ represent the expression levels of the *m*-th gene at the *k*-th measurements in the datasets $D_n^{+*}$ and $D_n^{-*}$, respectively. In this study, we also denote the weight parameters assigned to $\left( \left. {\textbf {X}} \right| _k^{+*}, \left. Y_n \right| _k^{+*} \right)$ and $\left( \left. {\textbf {X}} \right| _k^{-*}, \left. Y_n \right| _k^{-*} \right)$ as $w_k^{+*}$ and $w_k^{-*}$, respectively. Next, compute

6 $$\begin{aligned} W_n^+= &  \sum _{k = 1}^{|D_n^+|} w_k^{+*}, \end{aligned}$$

7 $$\begin{aligned} W_n^-= &  \sum _{k = 1}^{|D_n^-|} w_k^{-*}. \end{aligned}$$

If $W_n^+ \ge W_n^-$, find a value for $K^+$ such that $\sum _{k = 1}^{K^+} w_k^{+*} \ge W_n^-$ and $\sum _{k = 1}^{K^+ - 1} w_k^{+*} < W_n^-$, and set $K^-$ to $|D_n^-|$. Otherwise, find a value for $K^-$ such that $\sum _{k = 1}^{K^-} w_k^{-*} \ge W_n^+$ and $\sum _{k = 1}^{K^- - 1} w_k^{-*} < W_n^+$, and set $K^+$ to $|D_n^+|$.

This step is designed based on a bootstrap sampling. As our method considers the weight parameters $w_k^+$, $w_k^-$ and $w_k^0$, this step might seem to be complicated. When these weight parameters are all set to 1.0, for example, $W_n^+$ and $W_n^-$ represent the sizes of the sets $D_n^+$ and $D_n^-$, respectively, and $K^+$ and $K^-$, that are both set to $\min \left\{ |D_n^+|, |D_n^-| \right\}$, represent the numbers of examples used to compute a value for the function (8).

#### Step 5: Selection of Regression Tree

The random forest constructed by GENIE3 in Step 3 consists of $N_{subtree}$ regression trees, denoted as $\hat{G}_n^1, \hat{G}_n^2, \cdots , \hat{G}_n^{N_{subtree}}$. Select the optimal tree from these candidates and remove the others. This study quantifies the quality of tree $\hat{G}_n^i$ using the following function.

8 $$\begin{aligned} T_n(\hat{G}_n^i) = \sum _{k = 1}^{K^+} w_k^{+*} \times \max \left\{ -[\hat{G}_n^i (\left. {\textbf {X}}_{-n} \right| _k^{+*}) - \left. X_n \right| _k^{+*}], 0 \right\} \nonumber \\ + \sum _{k = 1}^{K^-} w_k^{-*} \times \max \left\{ [\hat{G}_n^i (\left. {\textbf {X}}_{-n} \right| _k^{-*}) - \left. X_n \right| _k^{-*}], 0 \right\} , \end{aligned}$$

where $\left. {\textbf {X}}_{-n} \right| _k^{+*} = (\left. X_1 \right| _k^{+*}, \cdots , \left. X_{n-1} \right| _k^{+*}, \left. X_{n+1} \right| _k^{+*}, \cdots , \left. X_N \right| _k^{+*})$ and $\left. {\textbf {X}}_{-n} \right| _k^{-*} =$ $(\left. X_1 \right| _k^{-*}, \cdots , \left. X_{n-1} \right| _k^{-*}, \left. X_{n+1} \right| _k^{-*} \cdots , \left. X_N \right| _k^{-*})$. As mentioned in section 3.1, we can use $G_n\left( {\textbf {X}}_{-n}\right) - X_n$ to estimate the sign of $\frac{dX_n}{dt}$. Therefore, the term $\max \left\{ -[\hat{G}_n^i (\left. {\textbf {X}}_{-n} \right| _k^{+*}) - \left. X_n \right| _k^{+*}], 0 \right\}$ will have a value of 0, for example, if the regression tree $\hat{G}_n^i$ correctly estimates the class label $\left. Y_n \right| _k^{+*}$ using the input vector $\left. {\textbf {X}} \right| _k^{+*}$. Otherwise, it will have a positive value. The function $T_n$ thus represents the degree of misclassification by the tree. Hence, the regression tree with the smallest $T_n$ value is determined to be optimal.

The aim of this step is to identify trees that correctly classify examples labeled ‘+’ and ‘−’. However, a problem of class imbalance can occur in actual classification tasks when the classifier must be trained using a fairly small number of examples in the minority class together with a large number of examples in majority classes. To prevent such a class imbalance from degrading the performance of the proposed method, this study employs the under-sampling approach (Chawla et al. 2002). Steps 4 and 5 of our method apply this approach.

#### Step 6: Conditional Branch

If the total number of regression trees selected thus far has reached $N_{tree}$, proceed to Step 7. Otherwise, return to Step 2.

#### Step 7: Computation of Confidence Values

Compute confidence values for regulations of the *n*-th gene from other genes by analyzing the ensemble of selected regression trees. Since the ensemble obtained in the previous steps has a structure equivalent to that of a random forest, we can compute confidence values for regulations using the same method employed in random-forest-based inference.

As described in Sect. 2.2, the original random-forest-based inference method (Kimura et al. 2019) uses the variance-reduction-based variable importance measure. However, for genetic network inference, the random-input variable importance measure has been shown to be more suitable (Kimura et al. 2022a). Therefore, the proposed method uses this measure to compute confidence values. Confidence values computed using this measure depend strongly on the random numbers used. To reduce the effect of random numbers, the confidence values are computed $N_{rnd}$ times using different random numbers, and their averages are used to rank the regulations.

### Remarks

Since the inference method proposed in this study uses GENIE3, sufficient gene expression data labeled ‘0’ are required for reasonable genetic network inference. While the proposed method cannot function without gene expression data labeled ‘0’, the method can infer genetic networks without data labeled ‘+’ or ‘−’. Note however that, when gene expression data labeled either ‘+’ or ‘−’ are unavailable, the proposed inference method becomes equivalent to GENIE3. Thus, our inference method can be regarded as yet another extension of GENIE3.

## Numerical Experiments with Artificial Data

To evaluate the performance of the proposed method, we conducted a series of experiments designed to confirm whether the proposed method can infer genetic networks from gene expression levels and the signs of their time derivatives.

### Construction of Training Data

In this study, we applied the proposed method to four artificial problems from the work done by Pratapa and colleagues (Pratapa et al. 2020): mCAD, VSC, HSC and GSD. These problems were designed to check the performances of methods that are capable of inferring genetic networks from single-cell gene expression data. The target networks of these problems consisted of 5, 8, 11 and 19 genes ($N = 5, 8, 11$ and 19), respectively, and were designed based on actual biochemical networks. Each of the problems contained 10 different datasets with 2, 000 cells. These datasets were constructed by solving a set of stochastic differential equations on the target network. In each of the problems, we performed 10 trials, each with a different dataset consisting of the measurements of 2, 000 cells. According to the following procedure, we constructed the training datasets for the inference method from each of the given datasets.

Pratapa and colleagues (Pratapa et al. 2020) assigned the pseudotime to each of the measurements in each of the given datasets. Based on this information, therefore, we constructed pseudo time-series datasets. Using local linear regression (Cleveland 1979), we smoothed each pseudo time-series dataset and then computed their slopes, which would be equivalent to the time derivatives of the gene expression levels if pseudotime were linearly proportional to actual time. Next, we assigned class labels to the measurements in the pseudo time-series data based on these computed slopes. When assigning class labels, however, we must consider the inherently high noise levels in single-cell gene expression data. For instance, when the slope of the smoothed expression level of the *n*-th gene at a pseudotime *pt* is positive, our study generally assigns the class label ‘+’ to the measurements at *pt* in the *n*-th subproblem. However, if the actual expression level of the *n*-th gene of a certain measurement at *pt* is higher than the smoothed expression level, it is unclear whether the expression is truly increasing. In such cases, we excluded that measurement from the training data for the *n*-th subproblem. Similarly, we also removed measurements in which the expression level of the *n*-th gene was excessively high or low, as we believe the genes in such cells were erroneously regulated.

**Fig. 1:**
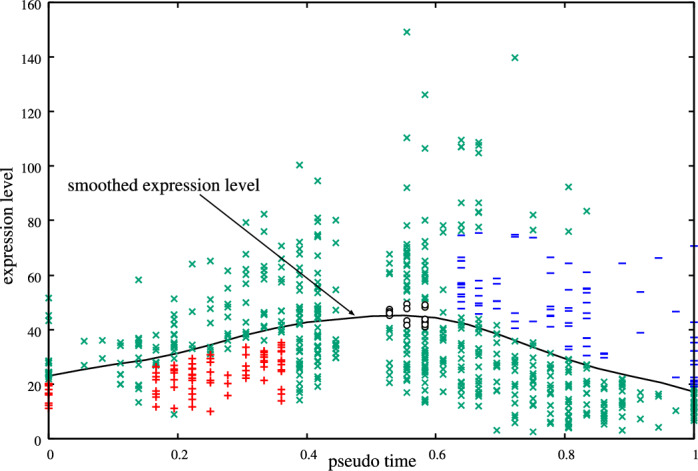
An example of class labels assigned to measurements in the pseudo time-series data. The figure shows the expression levels of a certain gene in the measurements and its smoothed expression level. Red ‘+’, blue ‘−’, black ‘$\circ$’ and green ‘$\times$’ symbols represent measurements labeled ‘+’, ‘−’, ‘0’ and ‘X’, respectively. These labeled data are used to infer regulations of the gene from the other genes

In this section, we denote the vector of gene expression levels for the *k*-th measurement having a pseudotime *pt* as $\left. {\textbf {X}} \right| _{pt}^k = (\left. X_1 \right| _{pt}^k, \left. X_2 \right| _{pt}^k, \cdots , \left. X_N \right| _{pt}^k)$, where $\left. X_m \right| _{pt}^k$ represents the expression level of the *m*-th gene in the *k*-th cell at *pt*. Based on the approach described above, we constructed the training data for the *n*-th subproblem corresponding to the *n*-th gene according to the following rule: if $\left. X_n \right| _{pt}^k$ falls within the top or bottom 5% of expression levels for the *n*-th gene across all cells in the pseudo time-series data, assign class label ‘X’ to $\left. {\textbf {X}} \right| _{pt}^k$, and thus exclude $\left. {\textbf {X}} \right| _{pt}^k$ from the training data; if $\left. X_n \right| _{pt}^k \le 0.9 \times \left. Sm_n \right| _{pt}$ and $\left. Sl_n \right| _{pt} > 1.2 \times \left. X_n \right| _{AVG}$, assign class label ‘+’ to $\left. {\textbf {X}} \right| _{pt}^k$; if $\left. X_n \right| _{pt}^k \ge 1.1 \times \left. Sm_n \right| _{pt}$ and $\left. Sl_n \right| _{pt} < -1.2 \times \left. X_n \right| _{AVG}$, assign class label ‘−’ to $\left. {\textbf {X}} \right| _{pt}^k$; if $0.9 \times \left. Sm_n \right| _{pt} \le \left. X_n \right| _{pt}^k \le 1.1 \times \left. Sm_n \right| _{pt}$ and $\left| \left. Sl_n \right| _{pt} \right| < 0.6 \times \left. X_n \right| _{AVG}$, assign class label ‘0’ to $\left. {\textbf {X}} \right| _{pt}^k$; in all other cases, assign class label ‘X’ to $\left. {\textbf {X}} \right| _{pt}^k$. Here, $\left. Sm_n \right| _{pt}$ is the smoothed expression level of the *n*-th gene at *pt*, $\left. X_n \right| _{AVG}$ is the average of the expression levels of the *n*-th gene in the pseudo time-series datasets, and $\left. Sl_n \right| _{pt}$ is the slope of the smoothed expression level of the *n*-th gene at *pt*. A sample of the assigned class labels is shown in Fig. 1.

It is not always an easy task to correctly estimate values for the slopes of the smoothed pseudo time-series data. As the proposed method relies only on the signs of the slopes, however, the slopes can be estimated without great precision. Even so, in order to infer reasonable networks, we must carefully estimate their values. Although we used the local linear regression (Cleveland 1979) in this study, we could use any smoothing technique. According to the procedure described above, we assigned the class labels to the measurements. If we can adjust the constant parameters used in this procedure according to the given data, we would infer more reliable networks. As described below, however, using the completely same labeling procedure, we successfully inferred several artificial and real networks. Whenever all pseudotimes assigned are between 0 and 1, therefore, this labeling procedure could be used without any modification.

As mentioned in Sect. 3.3, the proposed method requires sufficient gene expression data labeled ‘0’. Although Pratapa and colleagues (Pratapa et al. 2020) provided no dataset as steady-state one, the last parts of the pseudo time-series datasets seemed to consist of the cells measured under steady-state conditions. From each of the given datasets, therefore, this study constructed the steady-state dataset consisting of the cells to which the pseudotime greater than 0.85 is assigned. Each of these cells was assigned the class label ‘0’.

### Experimental Setup

Following recommended parameter values for the random-forest-based inference method (Kimura et al. 2019), we set the number of trees in the tree ensemble ($N_{tree}$) to 1, 000, the number of input variables to be considered at each internal node of each tree ($N_{test}$) to $\big \lceil \frac{N - 1}{3}\big \rceil$, and the maximum tree height ($N_{hmax}$) to 32. On the other hand, we set the number of trees in the random forest for GENIE3 used in the proposed method ($N_{subtree}$) and the number of iterations for computing variable importance scores ($N_{rnd}$) to 50 and 100, respectively, based on previous studies (Kimura et al. 2022b, 2023). The weight parameters $w_k^+$, $w_k^-$, and $w_k^0$ were determined using the established weighting method (Kimura et al. 2022a). We applied our proposed inference method to each of the four inference problems 10 times. Genetic networks were inferred using only the pseudo time-series gene expression data, the steady-state gene expression data, their associated class labels, and weight values.

### Results

In this section, we will quantify the inference method performance using the area under the precision-recall curve (AUPRC). The precision-recall curve of the method is obtained by calculating recall and precision at different confidence thresholds using the following equations:

$$ {\text{ recall }} = \frac{TP}{TP + FN}, \hspace{3mm} {\text{ precision }} = \frac{TP}{TP + FP}, $$

where *TP*, *FP*, and *FN* denote the numbers of true-positive, false-positive, and false-negative regulations, respectively. In this study, we computed recall and precision by constructing a regulatory network containing only regulations with confidence values exceeding a specified threshold, then comparing it with the gold-standard network. Next, we obtained the precision-recall curve of the algorithm by varying this confidence threshold. Auto-regulations and auto-degradations were excluded from the performance evaluation.

**Table 1:** Performances of the inference methods on the problems obtained from Pratapa and colleagues (Pratapa et al. 2020). AVG and STD represent the average AUPRC and its standard deviation, respectively. Median represents the median of the AUPRC values. ‘modified GENIE3’ refers to GENIE3 as used in the proposed method, incorporating the weight parameters $w_k^0$ and the random-input variable importance measure. ‘original GENIE3’ refers to GENIE3 without any modifications. ‘random selection’ represents the expected AUPRC value where the ranking of the regulations is randomly determined

	used data	mCAD	VSC	HSC	GSD
		AVG	AVG	AVG	AVG
		± STD	± STD	± STD	± STD
		Median	Median	Median	Median
proposed method	all measurements	0.63868	0.76510	0.60973	0.30946
		$\pm 0.06120$	$\pm 0.07665$	$\pm 0.04643$	$\pm 0.02295$
		0.64084	0.78616	0.61394	0.31622
modified GENIE3	measurements	0.60835	0.79205	0.50096	0.26106
(weights and riVIM)	labeled ‘0’ only	$\pm 0.04371$	$\pm 0.03515$	$\pm 0.02581$	$\pm 0.01110$
		0.61257	0.79676	0.50292	0.26264
original GENIE3	measurements	0.60789	0.80230	0.42831	0.27967
	labeded ‘0’ only	$\pm 0.04601$	$\pm 0.02821$	$\pm 0.02252$	$\pm 0.02130$
		0.60503	0.79717	0.42904	0.27905
modified GENIE3	all measurements	0.52782	0.63792	0.45226	0.25140
(weights and riVIM)	(used as labeled ‘0’)	$\pm 0.00746$	$\pm 0.06470$	$\pm 0.01127$	$\pm 0.00589$
		0.53105	0.64634	0.45410	0.25069
original GENIE3	all measurements	0.61446	0.66156	0.37759	0.24702
	(used as labeded ‘0’)	$\pm 0.01901$	$\pm 0.02466$	$\pm 0.01316$	$\pm 0.00466$
		0.61966	0.65503	0.38370	0.24592
random selection		0.65000	0.26786	0.23636	0.22222

The AUPRC values of the proposed method are listed in Table 1. We compared the performance of our proposed method with that of GENIE3 (Huynh-Thu et al. 2010). Since the original GENIE3 algorithm does not consider weight parameters and uses a variance-reduction-based variable importance measure to compute confidence values for the regulations, we also evaluated a modified version of GENIE3 that incorporates weight parameters ($w_k^0$) and uses the random-input variable importance measure. The table includes the AUPRC values for both the modified and original GENIE3. Although GENIE3 should infer genetic networks only using steady-state data in theory, it has been often used to analyze temporal data. The table also shows the performances of GENIE3’s that use all of the 2, 000 measurements as those labeled ‘0’.

As shown in Table 1, the proposed method outperformed the original and modified GENIE3’s at a 1% significance level on the HSC and GSD problems. Note that the statistical tests performed in this section were designed for comparing the means of two different groups (Welch’s *t*-test). The AUPRC values seemed to follow a distribution close to a normal distribution. Although the sample sizes were not always large, therefore, we could obtain reliable results from the statistical tests. The table also shows that the AUPRCs of the proposed method were better and worse than those of the best performers among the four GENIE3’s on the mCAD and VSC problems, respectively. However, the differences were not significant.

As mentioned previously, although GENIE3 should only use measurements labeled ‘0’ to infer genetic networks, researchers often use it to analyze measurements obtained under non-steady-state conditions. The table indicates that the removal of measurements obtained under non-steady-state conditions slightly improves the performance of GENIE3. In addition, the removal of non-steady-state data shortened the computation time of GENIE3. When the measurements labeled ‘0’ were only used, for example, the modified and original GENIE3’s averagely took about 4.98 minutes and 7.74 seconds, respectively, on a personal computer (Core i9-7960X; single-core used) to solve one of the 19 subproblems in the GSD problem. When all of the 2, 000 measurements were used as those labeled ‘0’, on the other hand, they required about 37.68 minutes and 41.53 seconds, respectively. Note that the higher computational cost of the modified GENIE3 is mainly caused by the use of the random-input variable importance measure (Kimura et al. 2022b). Although the proposed method trains a lot of regression trees, therefore, its computational cost was not too much higher than that of the modified GENIE3. In order to solve a subproblem in the GSD problem, the proposed method averagely took about 5.82 minutes.

While the performances of the proposed method were better than those of the random selection on the VSC, HSC and GSD problems, it was worse on the mCAD problem. However, this problem seemed to be difficult for inference methods. Pratapa and colleagues (Pratapa et al. 2020) applied 12 inference methods to the problems used in this section. Their experimental result showed that 8 of the 12 inference methods underperformed the random selection on the mCAD problem. From their result, we found that the proposed method ranked 5th among these inference methods with respect to AUPRC on the mCAD problem. Similarly, we also found that our method ranked 1st on the VSC, HSC and GSD problems. The target network of the mCAD problem is much denser than those of the others. As GENIE3 is reportedly not good at analyzing dense networks (Huynh-Thu et al. 2010), the proposed method might have the same drawback. As genetic networks are known to be sparsely connected (Thieffry et al. 1998), however, this drawback will not always hinder the use of our inference method.

## Numerical Experiments with Real Data

Next, we will evaluate the performance of the proposed method using actual single-cell gene expression data. In general, genetic network inference requires training datasets with significantly more measurements than genes. However, the actual gene expression datasets used in this study have a limited number of measurements. Therefore, we selected sets of genes that are known to work together and inferred the regulatory relationships among them.

### Analysis of MCF-7 Data

In this study, we analyzed single-cell gene expression data from MCF-7 (breast cancer cell line) cells subjected to continuous tamoxifen treatment (Magi et al. 2021). According to the original study, the MCF-7 cells began developing resistance to tamoxifen around week 5 or 6. Measurements were collected at weeks 0 (before tamoxifen treatment), 3, 6, and 9. Iida and Okada (2024) then clustered the cells into five classes: C1, C2, C3, C4, and C5. Our study was focused on the measurements for clusters C1, C2, C3, and C4, which are reportedly related to the development of tamoxifen resistance.

Through pseudo-temporal ordering analysis, Iida and Okada found that these cells developed along two different trajectories (Iida and Okada 2024). We hypothesized that the different trajectories of cell development reflect variations in gene regulation. Thus, we inferred two genetic networks, one for each trajectory.

#### Construction of Training Data

The original dataset consisted of 1, 000 measurements for 6, 082 genes (Iida and Okada 2024). We excluded 4, 743 low-activity genes having expression levels of 0 in many measurements. As mentioned previously, we used two pseudo time-series datasets, one constructed from gene expression data for the cells of clusters C1, C2 and C3 (648 measurements), and the other constructed from the cells of clusters C1, C2 and C4 (689 measurements). Each dataset corresponds to one of the trajectories mentioned above. Using local linear regression (Cleveland 1979), we smoothed each dataset and then computed their slopes. According the procedure described in Sect. 4.1, we then assigned class labels to the measurements in the datasets. For the steady-state data, on the other hand, we used the single-cell gene expression data measured at week 0, consisting of 326 measurements. Each of these measurements was assigned the class label ‘0’.

As mentioned previously, the data analyzed in this section were derived from MCF-7 cells treated with tamoxifen. To explicitly account for the effect of tamoxifen stimulation, we added an element to the gene expression data. The added element was set to 1.0 for measurements in the pseudo time-series data and to 0.0 for measurements in the steady-state data.

#### Experimental Setup

The objective of the experiments in this section was to confirm whether our proposed method could infer a reasonable genetic network from real single-cell gene expression data. To this end, we applied functional annotation clustering using DAVID (https://davidbioinformatics.nih.gov/) (Sherman et al. 2022) to the 1, 285 ($6,028 - 4,743$) genes remaining in our dataset. This yielded several sets of genes known to work together. We then used the proposed method to infer a genetic network among the genes in each selected set. Note that, in these experiments, we inferred the regulations of the selected genes from these genes and the added element using the steady-state dataset, either one of the two pseudo time-series datasets, and their class labels. All other experimental conditions were unchanged from those used in Sect. 4.2.

**Fig. 2:**
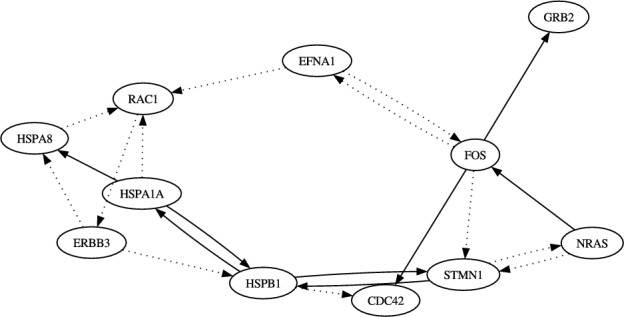
The network of the top 20 regulations obtained from the proposed method on the problem of the “signaling pathway” with the dataset from the clusters C1, C2 and C3. The solid lines represent the regulations recorded in the STRING database

**Fig. 3:**
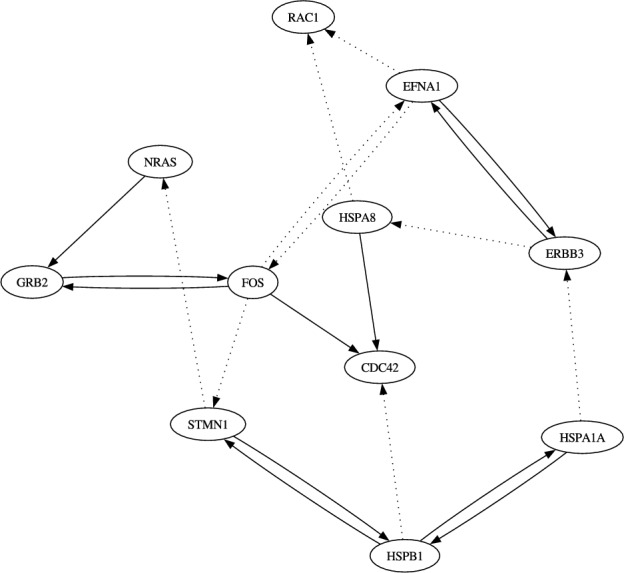
The network of the top 20 regulations obtained from the proposed method on the problem of the “signaling pathway” with the dataset from the clusters C1, C2 and C4

#### Inference of Networks of the Genes Related to “Signaling Pathway”

In this experiment, we selected 11 genes reported to be associated with “signaling pathways” (CDC42, EFNA1, ERBB3, FOS, GRB2, HSPA1A, HSPA8, HSPB1, STMN1, NRAS and RAC1) and inferred regulations among these genes and the added element ($N = 12$). Here, we inferred two genetic networks, each corresponding to one of the pseudo time-series datasets. Figs. 2 and 3 show the networks of the top 20 regulations with respect to the confidence values assigned by the proposed inference method on these problems. Due to the stochastic nature of this method, the confidence values varied slightly every trial. Therefore, we averaged the confidence values over 10 trials and ranked the regulations accordingly. Since the true structure of the target network was still unknown at this point, we compared our inferred regulations with known protein-protein interactions recorded in the STRING database (https://string-db.org/) (Szklarczyk et al. 2014). Unlike our method, which distinguishes directionality in gene regulation, the STRING database simply records interactions between proteins without indicating directionality. Thus, we ignored direction when comparing the inferred regulations with those in the database.

According to STRING, 8 of the top 20 regulations in the first trajectory and 11 in the second trajectory (indicated by the solid arrows in the figures) are supported by confirmed protein interactions in human and/or other species. Among the 121 possible candidates ($N \times (N-1) - (N - 1)$; self-regulations and regulations of the external stimulation were disregarded), a total of 44 ($22 \times 2$) regulations are recorded in the STRING database. Therefore, the expected number of matches when randomly selecting 20 regulations from the 121 candidates is approximately 7.27 ($20 \times 44 / 121$) on average. Note that the number of regulations recorded in the database within the 20 randomly selected regulations follows a hypergeometric distribution. Our experimental results thus indicate that the proposed method outperforms random selection.

Our results also show that plausible regulations with high confidence values obtained from the dataset of clusters C1, C2 and C3 were similar to those of clusters C1, C2 and C4, with the exception of two regulations: ERBB3 from EFNA1 and EFNA1 from ERBB3. While the regulation of ERBB3 from EFNA1 and that of EFNA1 from ERBB3 were ranked 16th and 19th, respectively, in the problem with the dataset of clusters C1, C2 and C4, they were ranked 59th and 42nd, respectively, in the problem with the dataset of clusters C1, C2 and C3. This suggests that these regulations cause different developments in MCF-7 cells. Further biological experiments would be valuable to confirm this hypothesis.

**Table 2:** Properties of the networks analyzed in the section 5.1

function	# of elements	# of regulations
of network	in network, *N*	recorded in database
signaling pathway	12	44
cell redbox homeostasis	16	82
cell body	14	68
regulation of cell cycle	16	54

**Table 3:** Summary of the experimental results from section 5.1.

network	pseudo time-series	# of reasonable regulations		
	data used	within top 20 regulations		
		proposal	GENIE3	random selection
signaling	C1, C2, C3	8	9	7.27
pathway	C1, C2, C4	11	8	7.27
cell redbox	C1, C2, C3	12	12	7.29
homeostasis	C1, C2, C4	12	11	7.29
cell body	C1, C2, C3	10	10	8.05
	C1, C2, C4	12	11	8.05
regulation	C1, C2, C3	11	9	4.80
of cell cycle	C1, C2, C4	6	8	4.80

#### Summary of Results of Experiments using MCF-7 Data

Using the MCF-7 dataset, we analyzed four small genetic networks, including the network described above. The properties of the target networks are shown in Table 2. For each network, we constructed two inference problems corresponding to the two gene expression trajectories. Thus, we solved eight inference problems in the experiments using MCF-7 data. The training data for each problem were constructed in the same manner as described in Sect. 5.1.1, and all experimental conditions were identical to those in Sect. 5.1.2.

Table 3 shows the numbers of plausible regulations among the top 20, as determined by the confidence values computed by both the proposed method and the modified version of GENIE3 on the eight problems. We ran the modified GENIE3 by applying the measurements labeled ‘0’ only. The table also includes the expected numbers of plausible regulations among 20 randomly selected candidate regulations for these problems. As shown, the proposed method consistently outperformed the random selection. Moreover, in 6 out of 8 cases, our method matched or exceeded the performance of GENIE3 in identifying plausible regulations. These results demonstrate that our method can reliably infer plausible genetic networks from actual single-cell gene expression data.

### Analysis of SAS Data

Lastly, to evaluate whether the proposed approach performs well on other single-cell gene expression datasets, we analyzed data from SAS cells (a human oral squamous cell carcinoma cell line) treated with TGF-$\beta$ (Takahashi et al. 2022). The dataset consisted of measurements of the SAS cells taken before and after treatment with TGF-$\beta$. Through pseudo-temporal ordering analysis, Takahashi and colleagues (Takahashi et al. 2022) revealed that SAS cells treated with TGF-$\beta$ differentiate into two types of mesenchymal cells: type 1 and type 2. As with the experiments described in section 5.1, we inferred two genetic networks, each reflecting a gene expression trajectory leading to one of the two mesenchymal cell types.

We obtained the single-cell gene expression data from the work done by Takahashi et al. (2022). From the original dataset containing 5, 595 measurements of 6, 000 genes, we excluded 4, 579 genes that showed expression levels of 0 in many of the measurements. For this experiment, we constructed two pseudo time-series datasets: one for the type 1 mesenchymal cells (2, 641 measurements) and one for the type 2 mesenchymal cells (4, 773 measurements). We also used the gene expression data measured before the treatment with TGF-$\beta$ (3, 184 measurements) as the steady-state dataset. We assigned class labels to the measurements in these datasets according to the procedure described in section 4.1. To explicitly account for the effect of TGF-$\beta$ treatment, we added an extra element to the gene expression data. The added element had values of 1.0 and 0.0 for measurements in the pseudo time-series and steady-state datasets, respectively.

Using DAVID (Sherman et al. 2022), we selected 16 genes associated with “mRNA splicing” and related processes (AKAP17A, CWC25, LSM10, LSM8, RBM5, FAM50A, NCBP2, PQBP1, SNRNP27, SART1, SART3, SFSWAP, THRAP3, ZNF326, FIP1L1 and ZNF638). We then inferred the regulations of these 16 genes from themselves and the added element ($N = 17$). When inferring a genetic network, we used the steady-state dataset, one of the two pseudo time-series datasets, and their corresponding class labels. All other experimental conditions were identical to those described in Sect. 4.2.

Of the top 20 regulations for both SAS cell trajectory problems, 4 (type-1 trajectory) and 4 (type-2 trajectory) seem plausible, as they are recorded in the STRING database (Szklarczyk et al. 2014) (see the supplementary files). Out of 256 candidate regulations ($N \times (N - 1) - (N -1)$), 46 are recorded in the database. Therefore, the expected number of plausible regulations in a random selection of 20 candidates is 3.59 ($20 \times 46 / 256$). By comparison, the numbers of plausible regulations identified by GENIE3 were the same as those of our method. These experimental results show that our proposed method did not underperform both random selection and GENIE3 in these SAS data problems. Although our method outperformed the random selection, the performance improvement was quite small in this problem. The small improvement might be due to the use of unreliable pseudo time-series datasets. It is important to note that the quality of the genetic networks inferred by our method depends heavily on the reliability of the pseudo time-series data produced through pseudo-temporal ordering analysis.

## Conclusion

To extract information from single-cell gene expression data, this study proposed a novel genetic network inference method. The proposed method utilizes both steady-state and pseudo time-series single-cell gene expression data for inference. Since precise timing information on measurements is not available in pseudo time-series data, our approach infers genetic networks using the signs of time derivatives of gene expression levels, which can be estimated from the data. We demonstrated the effectiveness of this method through numerical experiments using both artificial and actual gene expression datasets.

Many recently developed inference methods aim to improve reliability in genetic network inference by integrating information extracted from multiple types of data, such as gene expression data, TF-binding data, and chromatin accessibility data (Badia-i-Mompel et al. 2023). However, these methods often employ GENIE3 for extracting information from gene expression data. As discussed earlier, the proposed method can be viewed as an extension of GENIE3 that could be incorporated into these inference methods to extract more robust information from gene expression data.

A key advantage of the proposed method is its ability to utilize pseudo time-series data without relying on unnatural assumptions. However, as illustrated in Fig. 1, we excluded more than half of the measurements in the pseudo time-series data. In future studies, we aim to develop strategies to make more effective use of these excluded measurements.

## Supplementary Information

Below is the link to the electronic supplementary material.

Supplementary file 1 (pdf 88 KB)
