# Supplementary material for: Inference of Genetic Networks from Pseudo Time Series of Single-cell Gene Expression Data using Modified Random Forests
Source: Bull Math Biol. 2026 Feb 21;88(3):41. doi: 10.1007/s11538-026-01612-8 (PMC12923446; doi:10.1007/s11538-026-01612-8)
Supplement: Supplementary file 1 — (pdf 88 KB) [file 11538_2026_1612_MOESM1_ESM.pdf]

Supporting Information: Inference of Genetic Networks from Pseudo Time Series of Single-cell Gene Expression Data using Modified Random Forests

Shuhei Kimura, *et al.*
kimura@tottori-u.ac.jp

1 Weighting method

The performance of the random-forest-based inference method [1] can be improved by assigning appropriate values to the weight parameters. In order to determine these values, the weighting method has been proposed [2]. This method computes weight values based on the similarities between measurements. Although the weighting method was developed for the existing inference method [1], it can be used for the method proposed in this study. This section thus describes a way to compute weight values for the proposed method using this weighting method.

As mentioned in the sections 4 and 5 of the manuscript, our approach removes some measurements from the observed single-cell gene expression data. This study however performs the removal of measurements after the computation of weight values. Before computing weight values, the weighting method normalizes the given gene expression data so that the expression levels of each gene range from 0.0 to 1.0. Here, we represent the normalized measurements obtained from steady-state and pseudo time-series data as $\left.\overline{\mathbf{X}}\right|_1, \left.\overline{\mathbf{X}}\right|_2, \cdots, \left.\overline{\mathbf{X}}\right|_K$, where $\left.\overline{\mathbf{X}}\right|_k = (\left.\overline{X}_1\right|_k, \left.\overline{X}_2\right|_k, \cdots, \left.\overline{X}_N\right|_k)$, $\left.\overline{X}_m\right|_k$ is the normalized expression level of the m-th gene at the k-th measurement, and K is the total number of measurements. The weighting method then computes a weight value corresponding to the k-th measurement, w_k, according to

$$w_k = \left[\sum_{i=1}^{K} Sim\left(\left.\overline{\mathbf{X}}\right|_k, \left.\overline{\mathbf{X}}\right|_i \right) \right]^{-1}, \tag{1}$$

where

$$Sim(\mathbf{x}, \mathbf{y}) = \exp\left(-C \left| \mathbf{x} - \mathbf{y} \right|^2 \right), \tag{2}$$

$$C = \frac{C_w}{\text{median } S_{all}}, \tag{3}$$

$$S_{all} = \left\{ \left| \left.\overline{\mathbf{X}}\right|_i - \left.\overline{\mathbf{X}}\right|_j \right|^2 \middle| i, j = 1, \cdots, K, i < j \right\}, \tag{4}$$

and C_w (> 0) is a constant parameter. The recommended value for C_w is 15.

2 Random-input variable importance measure

The proposed method computes confidence values for regulations of the n-th gene from the other genes using the random-input variable importance measure [3]. The random-input variable importance measure quantifies the impact of an input variable on the output by measuring the fluctuation in output caused by changes in that variable. When using this measure, the proposed method computes the confidence value of the regulation of the n-th gene from the m-th gene, $C_{n,m}$, according to

$$C_{n,m} = \frac{1}{Sq} \left(WSE_R - WSE_0 \right), \tag{5}$$

where

$$WSE_R = \sum_{k=1}^{|D_n^0|} w_k^0 \left[\hat{G}_n^* \left(\left.\mathbf{X}_{-n}\right|_k^{(m)} \right) - y_k \right]^2, \tag{6}$$

$$WSE_0 = \sum_{k=1}^{|D_n^0|} w_k^0 \left[\hat{G}_n^* \left(\left.\mathbf{X}_{-n}\right|_k \right) - y_k \right]^2, \tag{7}$$

$$Sq = \sum_{k=1}^{|D_n^0|} w_k^0 (y_k - \overline{y}_{w0})^2, \tag{8}$$

$$\overline{y}_{w0} = \frac{1}{N_{w0}} \left[\sum_{k=1}^{|D_n^0|} w_k^0 y_k \right], \tag{9}$$

$$N_{w0} = \sum_{k=1}^{|D_n^0|} w_k^0, \tag{10}$$

$$y_k = \left. X_n \right|_k. \tag{11}$$

This section represents $D_n^0 = \left\{ (\left.\mathbf{X}\right|_k, \left.Y_n\right|_k) | k = 1, 2, \cdots, |D_n^0| \right\}$, and represents w_k^0 as the weight parameter assigned to $(\left.\mathbf{X}\right|_k, \left.Y_n\right|_k)$, where $\left.\mathbf{X}\right|_k = (\left.X_1\right|_k, \left.X_2\right|_k, \cdots, \left.X_N\right|_k)$. Note that, in order to compute confidence values, our method uses the measurements labeled '0' only. $\hat{G}_n^*$ is the approximation of function G_n, obtained by the proposed method. $\left.\mathbf{X}_{-n}\right|_k = (\left.X_1\right|_k, \cdots, \left.X_{n-1}\right|_k, \left.X_{n+1}\right|_k, \cdots, \left.X_N\right|_k)$. $\left.\mathbf{X}_{-n}\right|_k^{(m)}$ is a vector constructed by modifying the expression levels of the m-th gene in $\left.\mathbf{X}_{-n}\right|_k$. The expression levels of the m-th gene in $\left.\mathbf{X}_{-n}\right|_k^{(m)}$ is randomly drawn from the interval $[L_m, R_m]$, where

$$L_m = \min S_m, \tag{12}$$

Table 1: AUPRCs of the random-forest-based inference method on the mCAD, VSC, HSC and GSD problems. The performances of the proposed method are also shown.

	mCAD	VSC	HSC	GSD
	AVG<br>± STD<br>Median	AVG<br>± STD<br>Median	AVG<br>± STD<br>Median	AVG<br>± STD<br>Median
random-forest-based inference method	0.64885<br>±0.02800<br>0.65011	0.63329<br>±0.03944<br>0.64358	0.60011<br>±0.04093<br>0.58032	0.36422<br>±0.01208<br>0.36397
proposed method	0.63868<br>±0.06120<br>0.64084	0.76510<br>±0.07665<br>0.78616	0.60973<br>±0.04643<br>0.61394	0.30946<br>±0.02295<br>0.31622

$$R_m = \max S_m, \tag{13}$$
$$S_m = \left\{ X_m|_k \middle| k = 1, 2, \cdots, |D_n^0| \right\}. \tag{14}$$

Confidence values computed using the random-input variable importance measure depend strongly on the random numbers used. To reduce the effect of random numbers, the confidence values $C_{n,m}$'s are computed N_{rnd} times using different random numbers, and their averages are used to rank the regulations.

3 Experiments with random-forest-based inference method

While the random-forest-based inference method [1], that was basically designed for analyzing bulk-cell gene expression data, uses estimated time derivatives of gene expression levels, the proposed method uses their signs. Therefore, the proposed method tries to infer genetic networks using a smaller amount of information. When measurement time information is available, thus, we should not use the proposed method but use the random-forest-based inference method.

As pseudo time-series data lack precise temporal information about when the measurements were taken, it is difficult to precisely estimate time derivatives of gene expression levels from them. If the random-forest-based inference method [1] always infers reasonable genetic networks using imprecisely estimated time derivatives, however, the proposed method might be useless. In this section, we therefore applied the random-forest-based inference method [1] to the artificial and actual problems that were used in our manuscript, and checked its performance.

In this experiment, we assumed that pseudotime defined by the pseudo-temporal ordering analysis correlates linearly with actual time. We used $Sl_n|_{pt}$ as the time derivatives of the expression level of the n-th gene in the k-th

Table 2: The numbers of plausible regulations among the top 20 with respect to the confidence values computed by the random-forest-based inference method and the proposed method on the actual problems.

cell	network	pseudo time-series data used	# of reasonable regulations within top 20 regulations	
			RF-based method	proposed method
MCF-7	signaling	C1, C2, C3	8	8
	pathway	C1, C2, C4	6	11
MCF-7	cell redbox	C1, C2, C3	12	12
	homeostasis	C1, C2, C4	13	12
MCF-7	cell body	C1, C2, C3	10	10
		C1, C2, C4	7	12
MCF-7	regulation	C1, C2, C3	8	11
	of cell cycle	C1, C2, C4	5	6
SAS	mRNA splicing	type-1	2	4
	and related ones	type-2	4	4

measurement having a pseudotime pt, $\left.\frac{dX_n}{dt}\right|_{pt}^{k}$, where $Sl_n|_{pt}$ is the slope of the smoothed expression levels of the n-th gene at pt. We used the recommended values for the parameters of the random-forest-based inference method, and used the random-input variable importance measure [3] to compute confidence values of regulations. The other experimental conditions were the same as those of our manuscript.

The AUPRC values of the random-forest-based inference method on the four artificial problems are shown in Table 1. The table also shows the AUPRC values of the proposed method. As the table shows, the AUPRC values of the random-forest-based inference method were comparable to those of the proposed method on these problems. Note here that the proposed method infers genetic networks using a smaller amount of information. Therefore, erroneously estimated values for time derivatives of gene expression levels would degrade the performance of the random-forest-based inference method. There is no guarantee that reasonable values for time derivatives of gene expression levels can be estimated in the analysis of actual pseudo time-series data. The computational cost of the random-forest-based inference method was, on the other hand, much higher. The random-forest-based inference method and the proposed method averagely required about 50.0 minutes and 4.98 minutes, respectively, on the personal computer (Core i9-7960X) to solve one of the subproblems in the GSD problems.

Table 2, on the other hand, shows the numbers of plausible regulations among the top 20 regulations with respect to the confidence values computed by

the random-forest-based inference method and the proposed method on the actual problems. These results indicate that the proposed method slightly outperformed the random-forest-based inference method on the actual problems. Note here that, similar to the experiment described in the manuscript, we averaged the confidence values obtained by the random-forest-based inference method over 10 trials on the MCF-7 problems. Because of the high computational cost of the random-forest-based inference method, however, we used the confidence values averaged over three trials on the SAS problems. While the proposed method averagely took about 1.06 hours to solve one of the subproblems in the SAS problems, the random-forest-based inference method took about 73.2 hours to solve it. In this experiment, we then used the averaged confidence values to rank the regulations.

4 Networks inferred from real data

The networks of the top 30 regulations obtained by the proposed method on the problems with real data are shown in Figures 1-10.

References

[1] Kimura S, Tokuhisa M, Okada M (2019) Inference of genetic network using random forests: Assigning different weights for gene expression data. J. of Bioinformatics and Computational Biology 17: 1950015

[2] Kimura S, Sota K, Tokuhisa M (2022) Inference of genetic networks using random forests: A quantitative weighting method for gene expression data. Proc of 2022 IEEE Conf. on Computational Intelligence in Bioinformatics and Computational Biology: 123–130

[3] Kimura S, Takeda Y, Tokuhisa M, Okada M (2022) Inference of genetic networks using random forests: Performance improvement using a new variable importance measure. Chem-Bio Informatics J. 22: 88–109

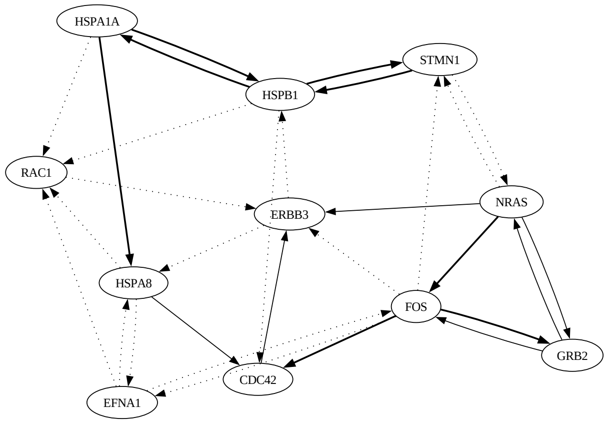

Figure 1: The network of the top 30 regulations obtained from the proposed method on the problem of "signaling pathway" with the measurements of the clusters C1, C2 and C3. Solid and bold lines represent the top 30 and 20 regulations, respectively, recorded in STRING database.

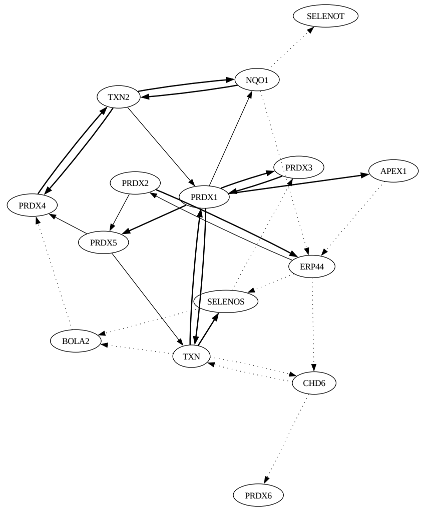

Figure 2: The network of the top 30 regulations obtained from the proposed method on the problem of "signaling pathway" with the measurements of the clusters C1, C2 and C4.

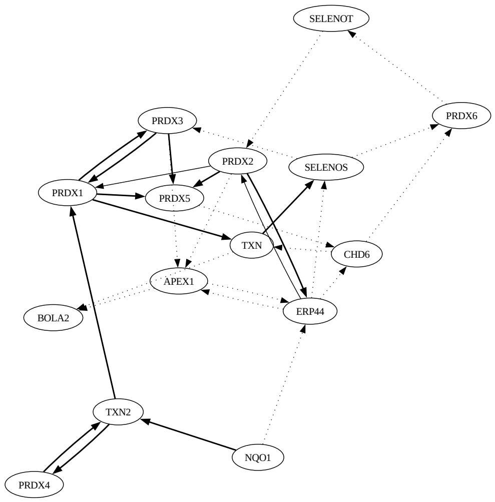

Figure 3: The network of the top 30 regulations obtained from the proposed method on the problem of "cell redbox homeostasis" with the measurements of the clusters C1, C2 and C3.

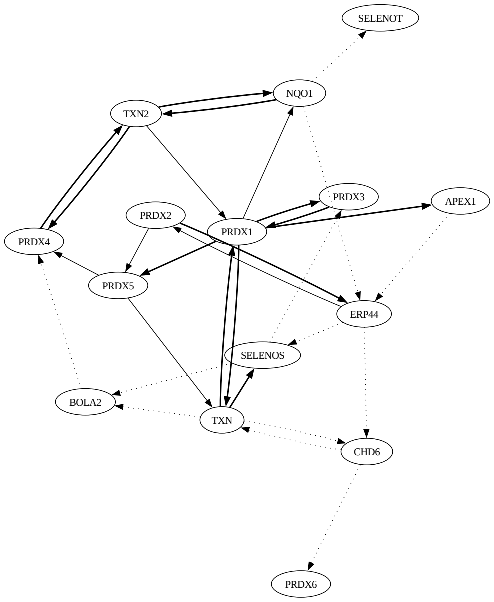

Figure 4: The network of the top 30 regulations obtained from the proposed method on the problem of "cell redbox homeostasis" with the measurements of the clusters C1, C2 and C4.

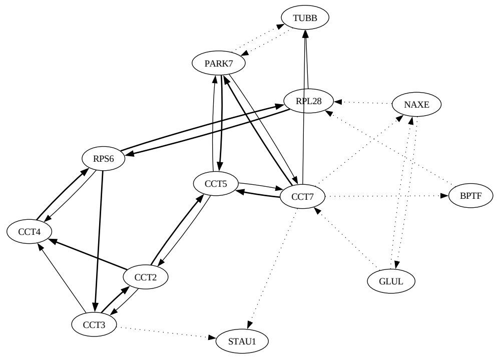

Figure 5: The network of the top 30 regulations obtained from the proposed method on the problem of “cell body” with the measurements of the clusters C1, C2 and C3.

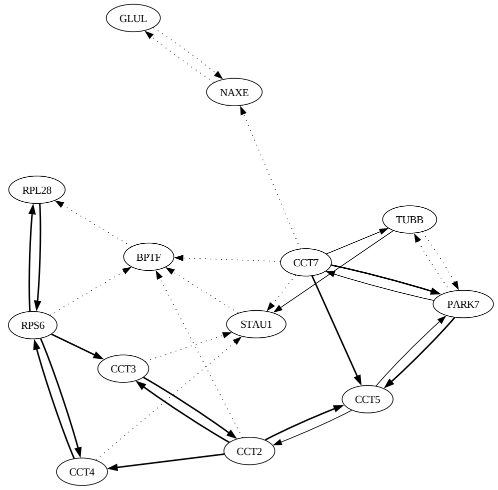

Figure 6: The network of the top 30 regulations obtained from the proposed method on the problem of “cell body” with the measurements of the clusters C1, C2 and C4.

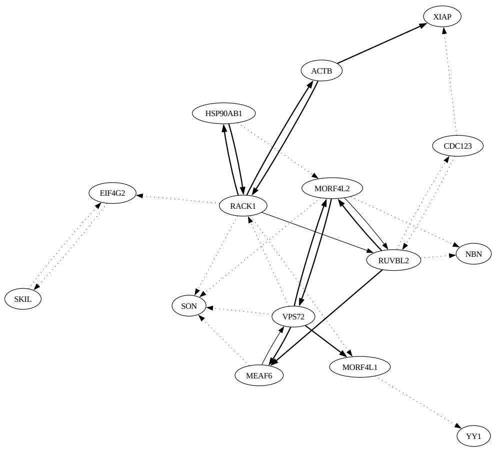

Figure 7: The network of the top 30 regulations obtained from the proposed method on the problem of "regulation of cell cycle" with the measurements of the clusters C1, C2 and C3.

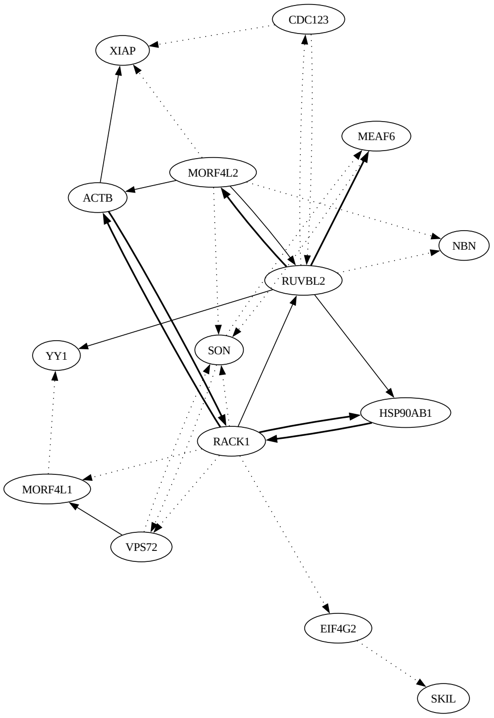

Figure 8: The network of the top 30 regulations obtained from the proposed method on the problem of "regulation of cell cycle" with the measurements of the clusters C1, C2 and C4.

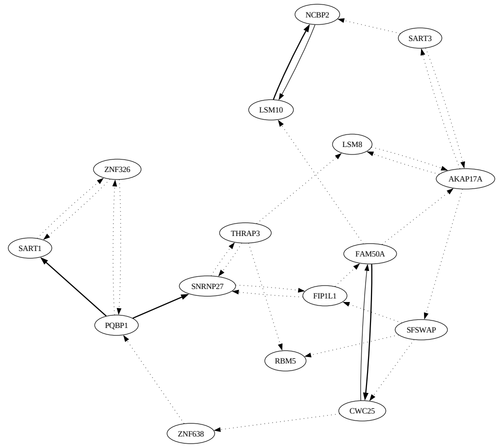

Figure 9: The network of the top 30 regulations obtained from the proposed method on the problem of type 1 mesenchymal cells with the SAS data.

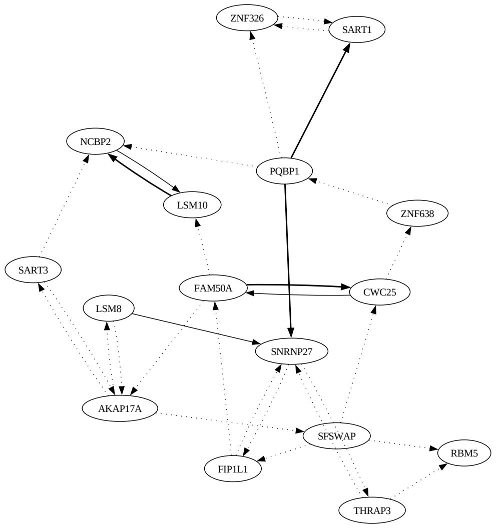

Figure 10: The network of the top 30 regulations obtained from the proposed method on the problem of type 2 mesenchymal cells with the SAS data.
